# Supplementary figures and images for: A Taxonomic Review of the Genus Telsimia Casey (Coleoptera, Coccinellidae) from China, with Descriptions of Eight New Species
Source: Insects. 2022 Sep 24;13(10):869. doi: 10.3390/insects13100869 (PMC9604187; doi:10.3390/insects13100869)

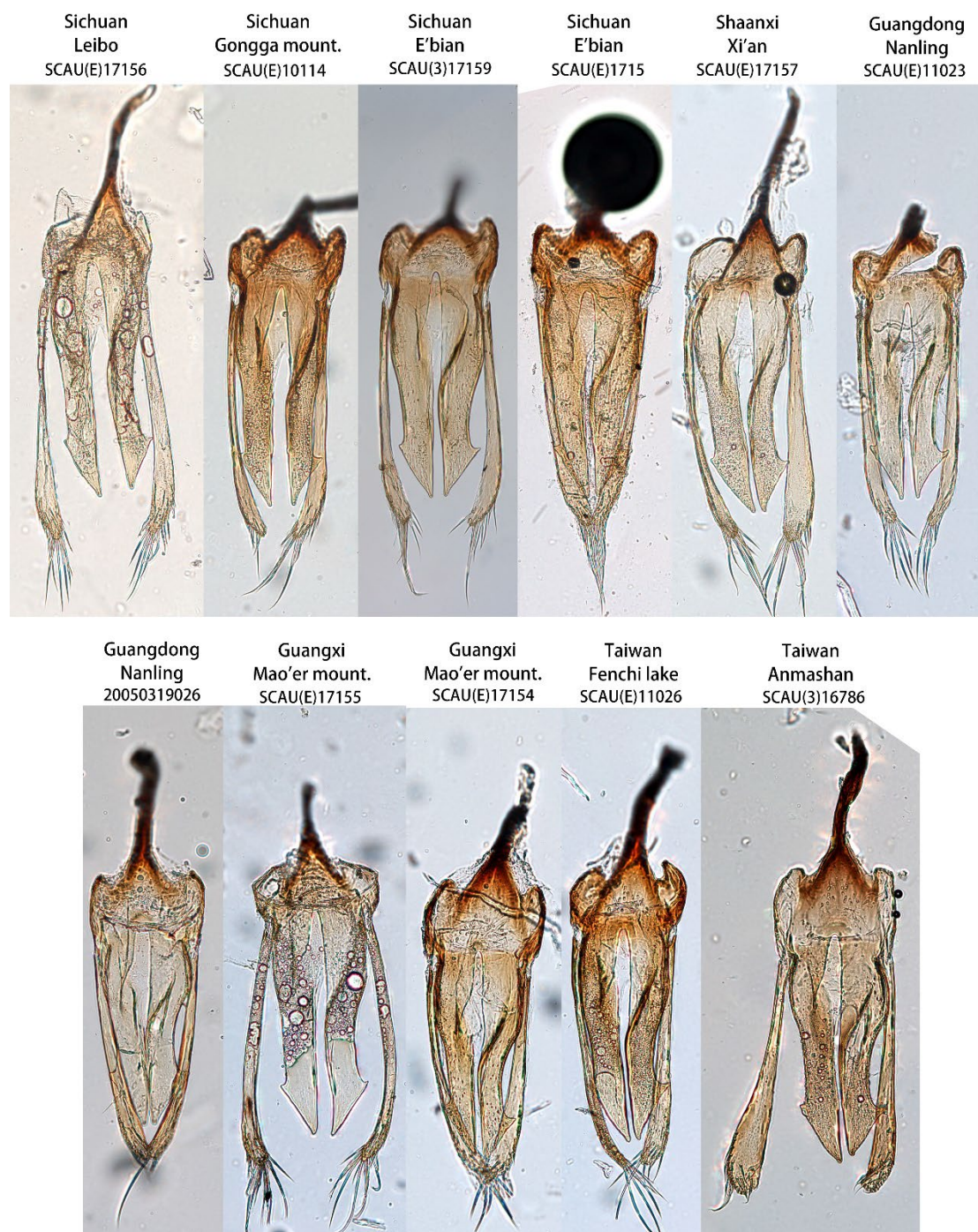

Figure S1. The tegmen of *Telsimia nigra* from different places in China

Supplement: Supplementary file 1 [file insects-13-00869-s001.zip › Figure S1-The tegmen of Telsimia nigra from different places in China.pdf]
